# How People Reason About *Stare Decisis*: The Role of Political Ideology

**DOI:** 10.3390/bs16071087

**Published:** 2026-07-01

**Authors:** Emily Saks, Eugene Borgida

**Affiliations:** Department of Psychology, College of Liberal Arts, University of Minnesota, Twin Cities, Minneapolis, MN 55455, USA

**Keywords:** motivated reasoning, political ideology, law, precedent, political psychology

## Abstract

Past research suggests that people may exhibit directional motivated reasoning whereby they are less skeptical of information that supports their political views. Recent U.S. Supreme Court decisions have elicited significant backlash, with some citing the fact that the decision overturned a longstanding legal precedent. *Stare Decisis* describes a core principle of the American legal system that courts are expected to adhere to past decisions, or precedent, when ruling on a case, unless there is an extraordinary reason(s) not to. To what extent, and by what process, does the public consider precedent in their reactions to such Court rulings? We investigate perceptions of precedent and the extent to which they are conditioned by the context of two real-world cases that broke with precedent: *Dobbs v. Jackson Women’s Health Organization* (2022) and the *Chevron* doctrine in *Loper Bright Enterprises et al. v. Raimondo, Secretary of Commerce, et al*. (2024). We find that ideological attitudes and attitudes toward precedent influence Americans’ attitudes toward upholding precedent, though their influence depends on the specific case being evaluated.

## 1. Introduction

In the past few years, several high-profile U.S. Supreme Court decisions have captured public attention. Among these include *Dobbs v. Jackson Women’s Health Organization* (hereafter referred to as *Dobbs*), decided in June of 2022, which overturned the Court’s previous ruling in *Roe v. Wade* (1973), granting Americans a constitutional right to abortion (Liptak, 2022). Others include rulings limiting colleges’ and universities’ ability to consider race in admissions (de Vogue et al., 2023) and the decision in *303 Creative LLC v Elenis* (2023) that ruled in favor of a Christian website designer who did not want to make wedding websites for gay couples. In the recent *Loper Bright Enterprises et al. v. Raimondo* (2024) case (hereafter referred to as *Loper*), the Court overturned the *Chevron* doctrine which states that when laws are unclear, federal agencies (and the experts in them) may fill in the details as long as they come up with a reasonable interpretation.

These cases have overturned past rulings of the Supreme Court, breaking with judicial precedent. The Latin phrase *Stare Decisis* describes a bedrock principle of the American legal system: that courts are expected to adhere to past decisions, or precedent, when ruling on a case. However, Supreme Court Justices continue to extol the virtues of precedent. Justice Neil Gorsuch, at his Senate confirmation hearing, for example, stated that: “Senator, precedent is the starting point for any good judge. Precedent is our history, our shared history, our patrimony; the wisdom of the ages, if you want to think of it that way. And it would be foolish of any judge to come in and think that he or she knows better than everybody who has come before them. It would be an act of hubris.” (*Confirmation Hearing on the Nomination of Hon. Neil M. Gorsuch to be an Associate Justice of the Supreme Court of the United States*, 2017, p. 215).

Other Supreme Court Justices have expressed concerns that overturning existing precedents might undermine public confidence in the perceived legitimacy of the Court. In fact, Justice Amy Barrett was reluctant to take on the *Dobbs* case at all, writing that “If the court’s opinions change with its membership, public confidence in the court as an institution might decline. Its members might be seen as partisan rather than impartial and case law as fueled by power rather than reason” (Kantor & Liptak, 2023, p. 59). It is certainly possible that when the Court appears to be rapidly changing its stance on issues, it may contribute to reduced public trust in the institution due to perceived political partisanship of its members, and more negative views of it overall. Indeed, the recent decisions have sparked debate and public outcry. Public opinion surveys show that favorable views of the Supreme Court have declined over the past few decades, with historic lows especially present among Democrats based on survey data from July 2023, around the time that several of these decisions first made the news (Pew Research Center, 2023). Research also reinforces the concern that Court decisions like *Dobbs* could lead to less public trust in the Court and an increase in perceptions that the Court is politically motivated (Levendusky et al., 2024).

While it is certain that the Court takes precedent into consideration, the question remains whether the public perceives this role of precedent and whether these views are moderated by peoples’ own political views. The purpose of the present study is to investigate how precedent, and individuals’ political ideologies (liberal, conservative) influence attitudes toward judicial decisions. To examine this question, we utilize a two-wave research design administered through the on-line platform, Prolific. In the first wave, participants were given a brief definition of precedent and asked for their initial attitudes about the idea, as well as responding to a variety of background and demographic questions. In the second wave, respondents were randomly assigned to one of three conditions. They read a statement demonstrating either *full support* for precedent, more *partial* support for precedent, or, in the *control* condition, they read nothing further about the concept of precedent and presumably made decisions based on their intuitive baseline attitudes toward precedent. The statements presented to participants were based on actual statements given by Supreme Court Justices on how precedent is implemented in the courts. Next, participants read about two recent cases: *Dobbs* (a well-publicized decision overturning precedent about Constitutional protection of abortion) and *Loper* (a less well-known decision overturning precedent about the role of federal agencies in public policy decision-making). Participants evaluated both cases and were asked whether, if it had been up to them, they would they have decided to uphold or overturn precedent. Finally, several questions were asked to assess evidence for motivated reasoning in participants’ evaluations.

If the public understands and considers precedent in their evaluations, we anticipated that they would be more likely to choose to uphold precedent in both cases, and that the effect would be stronger in the full support condition compared to the partial support or control conditions. If opinions about cases are primarily shaped by ideology, participants’ decisions about whether to uphold the decision will depend on whether the decision in the case is consistent with their own political views. For the *Dobbs* case, making decisions based on political views may mean being more likely to uphold precedent if they identify as politically liberal rather than conservative, as opposed to primarily relying on attitudes toward precedent. The *Loper* case offers a comparison, as public views may not be as crystallized on this case, given that it was less publicized in the media than *Dobbs*. Due to weaker political cues about which stance would be ideologically congruent or incongruent, participants may not have as clear an idea of which position to take when it comes to upholding or overturning precedent.

To our knowledge, no past research has experimentally examined how everyday Americans reason about precedent when evaluating recent Supreme Court decisions. However, in an era in which public trust in institutions such as the Supreme Court is low, and recent controversial Court decisions consistently make news headlines, it is important to investigate the general public’s knowledge of precedent and how they rely on their understanding of precedent and, potentially, their own political ideologies to reason about cases. In the next three sections, we briefly discuss how legal scholars regard precedent, the extent to which individual differences in political ideology may influence public construals of precedent, and the role of motivated reasoning when assessing judicial decisions.

### 1.1. Precedent

Gerhardt (2008) argues that precedent is a crucial principle of the law. In general, he argues, most areas of the law are settled, and overturning precedent is rare. Even in cases where they appear to overturn precedent, the weakening of those precedents may correspond with the strengthening of other precedents. Gerhardt argues that a precedent derives from a single decision that becomes more entrenched over time. The more a precedent is cited in a particular context, the more fixed its meaning becomes. A network of citations for a particular precedent allows it to exert a stronger restraining force on Justices’ future decisions.

Cases that are important for the legal field can also have greater recognition among the public, as is the case of *Dobbs*, which specifically relates to *Roe v. Wade* (1973), widely considered a landmark case. A network analysis by Fowler et al. (2007) found that *Roe v. Wade* (1973) ranks highly in terms of “outward relevance,” referring to cases which are frequently cited by other court decisions. This is in comparison to cases high in inward relevance which, in turn, cite many other decisions. They demonstrate that both inward and outward relevance contribute to the probability of judges citing that case in the future and its overall importance. An empirical study by Cross et al. (2010) found significant variance in individual Justices’ citation practices.

More recent theory by Staszewski (2023) argues that the Court has never articulated a coherent theory of precedence or identified specific conditions under which courts should follow or overrule past decisions, resulting in an inconsistent application of precedent, which invites criticism from the public, especially the critique that its decision-making is politically motivated. Staszewski (2023) suggests that, in practice, the principle of *Stare Decisis* cannot reliably be applied to promote a consistent, formal rule of law; however, rather than discarding it entirely, we should focus on its alternative uses. For example, he argues that *Stare Decisis* encourages deliberation, forcing courts to consider and respond to prior decisions from judges who may have had very different perspectives on the law.

In one of the few empirical studies on precedent, Black and Spriggs (2013) note that much past research suggests that older cases are generally cited less often than more recent ones and they investigated the rate at which Supreme Court precedents “depreciate” over time. Consistent with Gerhardt’s thinking, they found that precedents that have been legally interpreted more often by the Court depreciate more slowly than others. Overall, however, they find that precedents depreciate rather quickly, about 81% and 85% between their first and 20th years of age at the Supreme Court and U.S. Court of Appeals, respectively.

### 1.2. Examining the Role of Political Ideology in Views on Precedent

Overall, scholarship on precedent and *Stare Decisis* suggests that while precedent plays an important role in decision-making at the Supreme Court level, judicial decision-making is also influenced by political ideology in the form of “ideological voting” (Cross et al., 2010). One school of thought on precedent, for example, even suggests that the Justices first anchor on their preferred outcome, based on ideological considerations, and then seek out precedents to cite in support of that outcome. It is argued that this pattern of citation is merely a mask for the influence of ideology (Cross et al., 2010). However, there is far less empirical research on how the public thinks about these principles, especially the extent to which their thinking is influenced by their own political ideologies.

In one of the many studies on how the public perceives the Supreme Court, Malhotra and Jessee (2014) investigate how people’s political positions shape different types of support for the Court. Participants were asked how they would have voted on ten recent Supreme Court cases and how they thought the Court would vote on each case. Participants were also asked for their approval and trust in the Court as well as support for its institutional legitimacy (whether the Court has the right to say what the Constitution means even when the majority of the people disagree). The researchers also assessed the ideological position of all of the justices. In line with their hypotheses, they find that respondents whose views are most similar to the Court show the highest trust in it. Ideological distance was positively related to support for the Court’s counter-majoritarian function, meaning that those who are extreme exhibit greater institutional support for the Court.

Ultimately, their results suggest that individuals’ political ideologies might influence assessments of the Supreme Court. However, how might ideology and precedent interact to influence the way individual citizens judge court cases that have an established precedent?

One hypothesis that derives from the psychological literature on political ideology is that those who are politically conservative may show greater support for precedent, at least in their baseline attitudes. Predominant models of political ideology propose that it can be conceptualized as a single right–left dimension that contains two interrelated aspects: (a) promotion vs. resistance to social change and (b) acceptance vs. rejection of inequality, with conservatives having a greater tendency to resist change and accept social inequality (Jost et al., 2009). Political ideology is a motivated social cognition, meaning that it is believed to fulfill underlying psychological needs related to uncertainty and threat (Jost et al., 2009) that in turn drive social and political reasoning processes. Studies show that conservative ideology is tied to needs for managing various threats, including outward threats like terrorism, but also psychological threats like death anxiety.

Altogether, this research suggests that conservatives may display more of an attachment to the status quo, or the way things have been historically, as a way of preserving the status quo in society. Lammers and Baldwin (2018), in fact, show that conservatives express greater feelings of nostalgia. They also found that communicating liberal ideas by framing them as similar to the past (temporal focus) diminished ideological disagreement between liberals and conservatives on policies such as criminal justice, gun rights and diversity.

### 1.3. Motivated Reasoning

According to Bolsen and Palm (2019), motivated reasoning describes the process by which people evaluate and form judgements about new information. Research on motivated reasoning identifies two motivational processes that guide how people interpret information. One is *accuracy motivation*, which motivates people to evaluate information impartially and arrive at accurate conclusions. In contrast, people may be motivated to seek out and interpret information in ways that are consistent with their prior beliefs and worldviews. When anything other than an accuracy goal drives information processing, it is called *“directional” motivated reasoning*.

Building on seminal work on directional motivated reasoning by Kunda (1990), Taber and Lodge (2006) articulated a series of biases that people show in evaluating political information that operate mostly outside of conscious awareness and even when people are encouraged to be objective. For example, people tend to prefer to seek out information that aligns with their preexisting beliefs, evaluate arguments consistent with their beliefs as stronger than opposing arguments, spend more time reading attitudinally incongruent information and become more attitudinally extreme even when exposed to more balanced arguments.

Research in psychology and political science has continued to show how political ideology can lead to directional motivated reasoning. Partisans show biases in evaluation, being less skeptical of information that supports rather than challenges their political beliefs (Ditto et al., 2019). They also may show bias in information-seeking, preferring to seek out information in line with their beliefs, and the tendency may persist even when partisans are incentivized toward accuracy (Peterson & Iyengar, 2021). Derreumaux et al. (2022), in a series of studies, show that both evaluative bias (bias in evaluation of information) and sampling bias (bias in seeking out sources that align with preexisting beliefs) interact to influence consumption of information about political in-groups and out-groups.

There is debate as to whether people are capable of being objective in evaluating information, and under what circumstances this can occur. Studies by Redlawsk (Redlawsk, 2001, 2002; Redlawsk et al., 2010) suggest that accuracy can be enhanced under certain conditions. Groenendyk and Krupnikov (2021), for example, demonstrate that priming a more open-minded motivation can lead people to more actively process information and be more accepting of counter-attitudinal information.

Given the wealth of past research implicating political ideology in motivated reasoning, it is plausible that judicial decisions may reflect motivated reasoning processes. Badas (2016), for example, found that perceptions of U.S. Supreme Court decision-making follow a motivated reasoning pattern: respondents in a nationally representative random-digit telephone sample who approved of the Court’s decisions in two different cases perceived the Justices as motivated by their analysis of legal factors, whereas those who disapproved of the Court’s decisions “perceived the Justices as being motivated by non-legalistic factors such as their ideology, gender, religion, and partisanship” (p. 324). In other words, if the decision is approved, then it is perceived as legally sound; if not, then the decision is politically suspect. Badas (2023) extended this motivated reasoning logic to public perceptions of Supreme Court nomination proceedings: survey respondents who supported nominees preferred a legalized rather than a politicized nomination process. Such findings suggest that the public may view the Court, and the judiciary more generally, as *less* legitimate when rulings are contrary to their extant interests and attitudes.

In the current research, we seek to extend research on directional motivated reasoning to examine the extent to which it may play a role in understanding public perceptions of upholding or overturning longstanding legal precedents through the lens of political ideology.

### 1.4. The Present Study

There are few empirical studies on how the public reasons about precedent. This study may be the first (to our knowledge) to examine how everyday Americans think about precedent and how these views interact with their ideological beliefs when evaluating judicial decisions. The study will extend research on directional motivated reasoning to examine its influence in this context. This study was preregistered on OSF and can be found at https://osf.io/2q3mz.

#### Hypotheses

Our preregistered hypotheses were the following:

**Hypothesis** **1:** *We hypothesize that there will be a main effect of baseline views toward precedent, such that those who express higher support for precedent will be more likely to say that precedent should be upheld in both cases.*

**Hypothesis** **2:** *We predict a main effect of condition, such that those who are in the full support group compared to the partial support group will express greater support for upholding precedent in both cases.*

**Hypothesis** **3:** *We hypothesize that conservatives will be higher in baseline support for precedence compared to liberals across cases.*

**Hypothesis** **4:** *We hypothesize that there will be an interaction between views of precedent and ideology whereby conservatives will be more likely to say that precedent should be upheld in both cases, though this prediction will be attenuated when the decision conflicts with their ideological beliefs. Thus, we would expect conservatives to be more likely than liberals to uphold precedent in the Loper case, but less likely to uphold it in the Dobbs case, when doing so involves upholding a Constitutional right to abortion—a stance that is unpopular among conservatives.*

## 2. Materials and Methods

### 2.1. Pre-Test

A pre-test study (n = 129) was conducted using Prolific (Prolific Academic Ltd., 483 Green Lanes, London, England N13 4BS) to test participants’ understanding of the definition of precedent and their understanding of the case descriptions provided for *Dobbs* and *Loper*. After reading the case descriptions, participants were asked how easy or difficult it was for them to understand them and how legally technical they found them. These items were averaged to create a readability score. We found that the *Dobbs* summary had a readability score of 3.66 and *Loper* 2.82, which prompted us to make edits to the summaries. In the main study, the *Dobbs* summary had 3.70 readability on average and *Loper* had 3.29, demonstrating that these edits successfully improved the comparable readability of the two passages. However, we still included readability as a control in the main analysis due to potential comprehension differences. In addition, this finding encouraged us to include other controls that might relate to comprehension ability such as education.

On the pre-test, after providing their decision about whether they would uphold precedent in each case and how confident they were, there was an open-ended question asking participants to explain their reasoning. This was done to give us further insight into participants’ ability to reason about legal cases and the role of precedent. Two undergraduate research assistants coded the set of open-ended responses. Coding disagreements were resolved by consensus between the two coders. For *Dobbs*, discussion of precedent was the most commonly mentioned theme, followed by the idea of personal autonomy, and then by the mention of states’ rights. For *Loper* reasoning, the most common code had to do with confusion understanding the passage. However, discussion of the importance of precedent was still the fourth most commonly cited theme. These results further support the idea that people are capable of thinking about precedent in this context. Based on the most commonly mentioned themes, we crafted a multiple-choice question for the main study asking participants to identify a primary reason for their decision to uphold or overturn precedent. We also tested whether participants were able to guess the purpose of the study (examining the role of political ideology in evaluating legal cases) on the pretest. We found that it did not affect decisions to uphold or overturn the case, which gave us confidence that even if participants were able to deduce the purpose of the study, it did not affect the primary outcome variable. Of those who responded to the question asking them about the purpose of the study, only 45% were able to *partially* guess the primary objective, while no one *completely* guessed the primary research question.

### 2.2. Procedure

Participants who spoke English and were over the age of 18 were recruited through the online platform Prolific. At Wave 1, participants responded to a survey assessing their initial personal views of precedent, as well as their political affiliation and political ideologies. They were given a specific definition of precedent: “Precedent is a principle of the American legal system that when an existing law is challenged in the courts, that judges should make decisions about whether to overturn or uphold the law by referring back to past rulings on the same topic.” A definition was provided so all participants had a basic understanding of the concept prior to assessing their baseline views.

At Wave 2, participants were randomly assigned to the *full support*, *partial support* or *control* condition. In the full and partial conditions, they read statements from Supreme Court Justices on how the concept of precedent is used in legal decision-making, *either expressing full support for relying on precedent* or *a more conditioned (partial) stance.* In the *control* condition, participants did not read anything about precedent and presumably evaluated the cases based only on the basic definition provided at Wave 1. Right after, participants in all 3 conditions completed two manipulation checks: one question asking whether they recalled reading anything about precedent and another multiple-choice question asking them to describe their specific understanding of what they read. After the treatment, they read two passages, one summarizing the *Dobbs* case and the other summarizing the *Loper* case. The order in which they viewed the case summaries was randomized. They were asked whether they would have upheld or overturned precedent in each case, if it had been up to them, and several follow-up questions regarding how they evaluated the information in the case, including questions designed to assess motivated reasoning. Exact survey question wording can be found in the Supplemental Materials and on the study’s OSF page: osf.io/2q3mz.

### 2.3. Wave 1 Measures

*Baseline Attitudes Toward Precedent*: Participants were asked three items about their initial attitudes towards the idea of precedent. The first asks how important it is for judges to consider precedent when making decisions, and participants rated their response on a five-point scale. The second asks them the extent to which they agree or disagree with the statement that: “The strength of the law comes from following past precedents.” Finally, they rated their agreement with the statement that, “Relying on precedent is crucial to a society like ours that is governed by the rule of law.”

*Political ideology*: Participants were asked a single question to rate how liberal or conservative they are on a seven-point scale.

*Views on federal power*: Participants were asked “How much do you trust the federal government and federal agencies in Washington, D.C. to make the right decisions for the country?” Responses ranged from “none of the time/never” to “just about always.”

*Abortion identity*: Participants were asked a single question asking them to identify whether they consider themselves pro-choice, pro-life, or mixed/neither when it comes to the abortion issue. They were also offered a “Don’t know what the term means” option, in case they were unfamiliar with this terminology.

*Anti-intellectualism*: Participants were asked three questions used in past research on anti-intellectualism (Motta, 2018) to assess their belief in trusting common intuition over experts and intellectuals.

*Separate-Spheres Ideology (SSI)*. Participants were given the 15-item SSI scale (Miller & Borgida, 2016), which assesses the extent to which people endorse traditional gender roles and a gendered status quo. Items include statements such as “Women can learn technical skills, but it doesn’t come as naturally as it does for most men,” and “It’s natural for a woman to be fulfilled by taking care of her children, but most men feel better when they have a good career, too.” Participants rated their agreement with each item on a five-point scale.

*Approval of the court*: As in Malhotra and Jessee (2014), participants were asked, “How much do you approve of the performance of the Supreme Court?” (1 = strongly approve, 2 = somewhat approve, 3 = somewhat disapprove, 4 = strongly disapprove).

*Trust in the court*. As in Malhotra and Jessee (2014), participants were asked, “Do you agree or disagree with the following statement: The Supreme Court can usually be trusted to make decisions that are right for the country as a whole?” (1 = strongly agree, 2 = somewhat agree, 3 = somewhat disagree, 4 = strongly disagree)

*Political knowledge*. Participants were asked a standard set of 4 questions assessing participants’ knowledge of the political system, such as the length of the term of a U.S. senator and whose job it is to nominate judges to federal courts. These items were averaged together and entered into the models as a control.

### 2.4. Wave 2 Measures

*Participant assessment of the case:* Participants were asked, if they were a judge, how they would have decided the cases. They chose between upholding precedent or overturning precedent.

*Argument strength*: Argument strength is a common metric of directional motivated reasoning. Past research suggests that participants employing directional motivated reasoning tend to evaluate opposing arguments as weaker (Taber & Lodge, 2006). Therefore, we asked participants to rate the strength of the winning argument. For *Dobbs*, the winning argument was that there is no constitutional right to abortion, and for *Loper*, it was that courts should have more power to interpret laws relative to federal agencies.

*Evaluation time*: Evaluation time is also a common metric of directional motivated reasoning. Past research suggests that participants spend more time evaluating counter-attitudinal arguments when directional motivated reasoning is present (Taber & Lodge, 2006). Therefoe, our survey was programmed to record the amount of time participants spend on the page, from their first click to their last click on the page.

*Accuracy*: Like argument strength and evaluation time, accuracy of recall is a standard metric for assessing the presence of motivated reasoning. Participants were asked a series of True/False questions to examine their recollection of the case briefs they read about. If motivated reasoning is present, we expect that they will show lower scores on these items.

*Primary reason*: Participants were asked a multiple-choice question about the main reason for their earlier choice to uphold or overturn precedent in each case. The response options were created based on responses to an open-ended question from the pre-test which allowed us to identify the most common reasons cited by participants for their decision about precedent.

*Case familiarity*: Finally, we asked participants how familiar they were with each case before taking the survey. For *Dobbs*, about 51% of participants reported being at least moderately familiar with the case, while only 18% said the same for *Loper*.

*Readability:* In order to control for potential differences in participants’ abilities to comprehend the summary passages, we asked participants how easy or difficult the passage was for them and how legally technical they found it.

## 3. Results

In total, 400 participants were recruited to take part in Wave 1 of the study. After removing those who failed the captcha (presented immediately after participants read the consent form) and those who failed the attention check, there remained 393 valid responses on Wave 1. The attention check was after the consent, captcha, and after the participant entered their Prolific ID number. For Wave 2, we had 330 valid responses. We examined the sample characteristics of those retained in the sample from Wave 1 to Wave 2 compared to those who dropped out of the study after Wave 1. The two samples were comparable on key demographics such as political ideology, political knowledge, views toward precedent, political knowledge, SSI, age and education. See the Supplemental Materials for comparisons.

We also included two manipulation check items asking participants (1) whether they recalled reading anything about precedent directly after the manipulation and (2) a question assessing their comprehension of what they read. We excluded participants in the full or partial conditions who responded (incorrectly) that they did not read anything about precedent. As for the second question, 75% of participants in the full condition gave the correct response (“Precedent is very important and should almost always be upheld unless there is a compelling legal reason not to”) and 73% of participants in the partial condition gave the correct response (“Precedent is a starting point, but judges should consider other factors that might have changed since the original law was made”). Participants in the full condition who did not select “Precedent is very important and should almost always be upheld unless there is a compelling legal reason not to” or participants in the partial condition that did not select “Precedent is a starting point, but judges should consider other factors that might have changed since the original law was made” were excluded. This left us with a final sample size of 270. We did not exclude participants in the control group based on whether they accurately recalled reading about precedent because participants were close to evenly split on whether they answered “Yes” or “No” to this question and the second question did not apply in the control condition because participants had not read anything about precedent. This resulted in 111 participants in the control condition, 83 in the full condition, and 76 in the partial condition. These manipulation checks combined with the earlier attention check gave us confidence that the sample largely was paying attention to the survey material and understood what they had just read about precedent.

As for the sample, 45% identified as conservative-leaning (selected “slightly conservative,” “conservative,” or “very conservative”) and 49% identified as liberal-leaning (selected “slightly liberal,” “liberal,” or “very liberal.”). 21% of the sample identified as Black, 66% as White and 12% were categorized as “other,” a category that included mixed-race, Hispanic, Asian/Asian-American and Indigenous people because there were too few in each of those categories to analyze them separately. There was a range of educational attainment, with 8% completing high school, 23% completing some college or an associate’s degree, 45% completing a four-year college degree and 24% completing a postgraduate degree. As the sample leaned more highly educated, we included education as a control in all our analyses. For age, 23% were 18–29, 49% were 30–49, 19% were 50–64 and 9% were 65 or older.

As in the pretest, we saw evidence that participants did explicitly consider precedent in their reasoning. On the multiple-choice question asking about their reasoning process, crafted based on the open-ended responses from the pretest, 131 participants mentioned personal choice, 64 participants explicitly mentioned favoring upholding precedent, and 53 participants mentioned state regulation over federal regulation when it came to the *Dobbs* decision. 22 participants did not respond or cited some other reason. For *Loper*, 85 identified being concerned about the balance of power in the government as the main reason for their decision, 62 explicitly discussed upholding precedent, 61 mentioned greater regulation on specific policy issues, and 35 mentioned being specifically concerned about the responsibility of fishing companies to pay for the monitors in the case. The rest did not respond, cited another reason or expressed difficulty understanding the material.

All statistical analysis were done in R (version 4.6.0).

### 3.1. Baseline Views Toward Precedent: Liberals vs. Conservatives

We hypothesized that conservatives may be higher in baseline support of precedent. To test this, we conducted a Wilcoxon test (nonparametric version of a t-test comparing two group means) investigating whether support for precedent differs between liberals and conservatives. Liberals (M = 3.925) and conservatives (M = 3.975) showed no significant differences in baseline views toward precedent (W = 7558.500, *p* = 0.593).

### 3.2. Effect of Baseline Views Toward Precedent, Condition and Ideology on Choice to Uphold Precedent

#### 3.2.1. Dobbs Case

We fit a logistic regression model with the choice to uphold or overturn precedent in the *Dobbs* case (upholding the *Roe* precedent and siding against Jackson Women’s Health Organization) as the outcome variable. This model included baseline precedent views, experimental condition, political ideology (coded as either liberal or conservative), and the interaction between political ideology and baseline views. Political ideology was coded as either liberal or conservative to make interpretation as to whether one’s political ideology would be generally pro or counter-attitudinal to the decision in the case, which was particularly relevant for *Dobbs*. We added education, gender, and political knowledge as demographic controls. We also included the readability score as a control, to adjust for possible differences in comprehension of the materials and trust in the Supreme Court as a variable that could potentially influence participants’ evaluations. Finally, we included abortion identity (pro-choice or pro-life), and Separate-Spheres Ideology as theoretically relevant controls related to abortion attitudes. McFadden’s Pseudo R-squared for the model was 0.394, indicating strong model fit.

Abortion views assessed at Wave 1 emerge as the most significant predictor of decisions to uphold the *Dobbs* precedent (B = −1.880, *p* < 0.001), with Separate Spheres Ideology also playing a significant role (B = −0.693, *p* = 0.034), indicating that those who are more pro-life and those who more strongly endorse the gendered status quo are less likely to uphold precedent (see Table 1). Since coefficients represent log odds, the interpretation of the −1.880 coefficient is that people who are pro-life had 0.153 times greater odds of upholding precedent, holding all other variables constant. We failed to find significant main effects of baseline views toward precedent, political ideology or a significant interaction between them on the decision to uphold precedent. One reason for our failure to find significant results may be due to multicollinearity, as the ideology variable and its interaction with baseline views specifically exhibited high multicollinearity with other variables in the model (see Supplemental Materials for the full VIF table). As ideology was crucial to our pre-registered hypotheses and analysis plan, we could not remove it. Though we failed to find the specific main effects and interactions hypothesized, our results suggest that ideological factors broadly may influence people’s evaluations of the *Dobbs* case. In our preregistration, we listed abortion identity and SSI as exploratory variables to include in relation to *Dobbs,* and they were tested as exploratory in these models. We exploratorily tested a second interaction between ideology and condition, but it was not significant for either *Dobbs* or *Loper*.

#### 3.2.2. Loper Case

We fit a logistic regression with baseline views of precedent, experimental condition, political ideology (coded as liberal or conservative), and the interaction between baseline views and ideology, as we did for the *Dobbs* case. We then added in the same demographic controls as used in the *Dobbs* model, as well as readability score, and trust in the Supreme Court, as before. We also included several theoretically relevant controls related to the content of the *Loper* case; specifically, anti-intellectualism and views on federal power. McFadden’s Pseudo-R-squared was 0.152 for the model. With controls added, baseline views toward precedent are marginally significant in predicting the decision to uphold precedent in the *Loper* case (B = 0.740, *p* = 0.046), with those who show greater support for the idea of precedent at Wave 1 being more likely to uphold the decision in the context of the *Loper* case (see Table 2). Trust in the Supreme Court (B = −0.620, *p* = 0.017) was also significant, with *higher* trust being associated with less likelihood of upholding precedent.

We failed to find a significant main effect for condition, ideology or the interaction between baseline views and ideology as hypothesized. Again, political ideology and the interaction term exhibited multicollinearity, which may explain the results. Though the results do not specifically align with the hypothesized main effects and interactions, the fact that baseline views toward precedent and variables related to legal reasoning (e.g., Trust in the Court) are significant predictors for the *Loper* case, but not the *Dobbs* case, suggests that ideology may play a greater role in reasoning about the more politically charged content of the *Dobbs* case as opposed to the more obscure, bureaucratic content of the *Loper* case. In our pre-registration, we indicated that we wanted to explore how trust in the Court, anti-intellectualism and views toward the federal government might be related to the *Loper* case as exploratory measures and later included them in the models as moderators.

### 3.3. Evidence for Motivated Reasoning

#### 3.3.1. Accuracy Index

The accuracy index was the first of three measures of motivated reasoning included post-experimentally. We expected that those engaging in more ideological motivated reasoning would be more likely to misremember certain details of the case summaries, resulting in lower accuracy scores. We used linear regression to examine the effect of baseline views, condition and ideology on accuracy scores for the *Dobbs* case, with the same controls and interaction as in Section 3.2.1. Only political knowledge (B = 0.415, *p* = 0.094) and gender (B = 0.181, *p* = 0.082) approached significance (see Table 3). We tested an exploratory interaction term between ideology and condition, but it was not significant.

We repeated the same analytic strategy for the accuracy scores for *Loper*, examining the role of baseline views, condition and ideology with interaction and controls. Only political knowledge was significant (B = 0.720, *p* = 0.011), meaning that 1-unit increases in political knowledge are associated with an average increase of 0.720 in accuracy score (see Table 4). We tested an exploratory interaction term and found that accuracy scores were slightly higher in the full condition relative to the control (B = −0.657, *p* = 0.018).

#### 3.3.2. Evaluation Time

Next, we examined evaluation time for evidence of motivated reasoning, as research suggests that people spend longer evaluating counter-attitudinal information. We examined the effect of baseline views of precedent, experimental condition, ideology and the interaction between baseline views and ideology on time spent evaluating the *Dobbs* case. The effect of being assigned to the partial condition (B = −57.657, *p* = 0.048) relative to the full and control conditions was marginally significant, with those in the partial condition possibly taking slightly less time in evaluating. However, abortion identity (B = 97.993, *p* = 0.002) and political knowledge (B = 192.086, *p* < 0.001) emerged as stronger predictors of evaluation time (see Table 5). Specifically, those who were pro-life spent more time evaluating the case description than those who were pro-choice, contrary to what was expected, and those high in political knowledge also took more time evaluating.

For *Loper* evaluation times, the effect of the full condition was significant (B = 66.154, *p* = 0.036), with longer times than the control condition (see Table 6). We tested an exploratory interaction between ideology and condition, and it was not significant for evaluation time for either case. Overall, there was no consistent evidence for motivated reasoning as operationalized with evaluation time.

#### 3.3.3. Argument Strength

Finally, we examined evidence for motivating reasoning as operationalized by differences in ratings of argument strength. We expected that liberals might rate the strength of the arguments provided in the *Dobbs* case as weaker than conservatives because the decision was more likely in conflict with their ideological views. We considered that this may be different from the *Loper* case where the case facts were less well-known and perhaps seen as more arcane and, therefore, participants may have been more unsure about what stance to adopt.

We used a chi-square test of independence to examine the relationship between ideology (liberals vs. conservatives) and argument strength ratings of the *Dobbs* case (not at all strong, somewhat strong, moderately strong, very strong, extremely strong). The results indicate convincing significant differences (χ^2^ = 82.508, *p* < 0.001). As predicted by directional motivated reasoning logic, 57% of conservatives said the *Dobbs* argument was extremely or very strong, compared to only 12% of liberals (see Table 7).

For the *Loper case*, there were also differences in argument strength by ideology (χ^2^ = 19.141, *p* = 0.001). 39% of conservatives rated the *Loper* argument as extremely or very strong, while only 27% of liberals did the same (see Table 8). Conservatives seemingly are more supportive of the *Loper* decision, shifting more decisional authority to the courts as opposed to experts within scientific agencies, than liberals.

## 4. Discussion

In one of the first empirical studies on this topic, we investigated how the public perceives the idea of legal precedent in the context of two very different SCOTUS decisions involving well-established legal precedents. Specifically, we examined whether (1) liberals and conservatives differ in baseline support for upholding precedent, (2) whether political ideology and baseline views toward precedent influence and/or interact to predict support for upholding or overturning precedent in the two cases, and (3) whether there was evidence for motivated reasoning in the decision-making process for average Americans when thinking about precedent in these two cases.

Contrary to what was expected, we did not find support for the idea that liberals and conservatives differ in baseline support for upholding precedent in these two cases. Even though conservatives might have a greater attachment to the past, it apparently does not uniformly predict support for upholding past precedents, at least in the two cases used in this study. When it comes to understanding the roles of ideology and views toward precedent in predicting Americans’ support for precedent in *Dobbs* and *Loper,* we found that ideological factors broadly influence decision making, though we were unable to separate specific ideological factors due to multicollinearity. This finding is also in line with past research on *judicial* decision-making, suggesting that factors other than precedent influence how Justices make their decisions (Cross et al., 2010).

When it came to the effect of baseline attitudes about precedent, such attitudes marginally predicted upholding precedent in *Loper* but not *Dobbs.* Though it was not consistent across the two cases, as hypothesized, the importance of precedent in predicting *Loper* decisions is generally consistent with our expectation that people would be more likely to rely on precedent when the case was more unfamiliar or involved case facts that were more arcane. Similarly, the role of ideological variables such as SSI and abortion identity (being pro-choice or pro-life) in having a greater influence in decisions to uphold precedent in relation to *Dobbs* compared to *Loper* is in line with this prediction. However, we failed to find support for a consistent main effect of baseline views toward precedent (H1), condition (H2) or the significant interaction between political ideology and baseline views toward precedent (H4) across cases.

We included several measures to test for the presence of motivated reasoning, and in both cases, we expected to find evidence of motivated reasoning using these conventional metrics for assessing the influence of motivated reasoning. Neither the memory accuracy items nor the evaluation time measure provided support for the role of motivated reasoning. However, political knowledge influenced both of these measures, which could suggest that they reflect participants’ interest in politics and attention to the subject matter rather than ideologically motivated reasoning. Importantly, in our results for argument strength ratings, we found that conservatives did rate the winning argument in *Dobbs* as stronger than did liberals, and that the ideological difference in ratings was larger for *Dobbs* (where political cues are stronger) compared to the *Loper* case. Thus, conservatives did perceive the *Dobbs* argument to overturn abortion as stronger than did liberals, which is in line with our general expectation that political ideology would influence perceptions of this case in a motivated manner. Interestingly, we also found that conservatives rated arguments higher in the *Loper* case as well (to a lesser degree than for *Dobbs*). There are at least two speculative explanations for conservatives’ tendency to rate arguments as stronger in both cases than liberals. One is that in the *Dobbs* case, higher ratings may have been due to the decision aligning with their pro-life views; but in the *Loper* decision higher ratings may be because conservatives believe that courts having greater interpretive power will result in decisions that align more with their beliefs. Another possible explanation is that conservatives may also rate the Loper arguments more highly because they believe that the courts, and not scientific experts in federal agencies, may be more inclined to rule in favor of upholding the status quo.

Other limitations were present in this line of research. One concerning limitation was the fact that our manipulation was valid but perhaps insufficiently strong to induce changes in participants’ attitudes toward precedent. Because of this, we did not find many significant effects for experimental condition and were unable to establish whether manipulating precedent as a decision rule caused effects on the decision to uphold precedent in the two cases. By contrast, entrenched and stable individual differences in gender ideology (as measured by Separate Spheres Ideology) and abortion identity (pro-choice vs. pro-life) were more influential in predicting whether precedent was upheld or overturned. In addition, standard measures used to assess the influence of motivated reasoning showed inconsistent effectiveness at assessing motivated reasoning in this context, so future research should explore how best to assess the extent to which motivated reasoning (or some other explanation of the process involved) was the key driver of effects. Finally, our results relied on an online sample of participants and self-report measures and therefore may not generalize to all Americans.

Overall, our results demonstrate that a diverse sample of the American public is most certainly capable of reasoning about legal precedent and using precedential thinking to inform their evaluations of cases. But, in this research, domain-specific individual differences rather than political ideology may have a greater role in shaping respondents’ attitudes about upholding or overturning precedent in two prominent Supreme Court decisions. Replication of these findings with other cases where even longer-standing precedent has been overturned (e.g., *Kennedy v. Bremerton School District*, 2022) would be an obvious next research step. The factors that shape Americans’ attitude toward upholding or overturning precedent in Supreme Court decisions may well depend on the specific case in question. Additionally, the extent to which ideology and views toward precedent influence average citizens’ evaluations of legal cases could be examined outside of constitutional and statutory law contexts and in legal systems that differ from the American system, which heavily weights precedent.

## Figures and Tables

**Table 1:** Logistic regression model predicting whether participants chose to uphold precedent in the *Dobbs* case (as opposed to overturning it). Coefficients represent the log-odds of upholding precedent in the *Dobbs* case. Condition, readability, and choice to uphold were measured on Wave 2, while the other controls were measured on Wave 1.

Variable	β	SE	Z	*p*
Intercept	3.254	2.019	1.612	0.107
Baseline Precedent Views (W1)	0.384	0.373	1.029	0.304
Political Ideology, liberal	4.563	3.798	1.201	0.230
Condition, full	0.428	0.513	0.834	0.404
Condition, partial	0.449	0.529	0.849	0.396
Education, some college/associate’s	0.390	0.578	0.674	0.500
Education, four-year degree	0.405	0.519	0.781	0.435
Education, postgraduate	0.800	0.459	1.743	0.081
Gender, woman	−0.551	0.445	−1.237	0.216
Political Knowledge	0.579	0.968	0.598	0.550
Court Trust	−0.310	0.333	−0.931	0.352
Abortion Views, pro-life	−1.880	0.526	−3.571	<0.001 ***
SSI	−0.693	0.326	−2.124	0.034 *
Readability score	−0.134	0.294	−0.456	0.648
W1 Attitudes: Political Ideology (liberal)	−0.682	0.944	−0.723	0.470

*Note*. * *p* < 0.05; *** *p* < 0.001.

**Table 2:** Logistic regression model predicting whether participants chose to uphold precedent in the *Loper* case (as opposed to overturning it). Coefficients represent the log-odds of upholding precedent in the *Loper* case. Condition, readability, and choice to uphold were measured on Wave 2, while the other controls were measured on Wave 1.

Variable	β	SE	Z	*p*
Intercept	1.553	1.713	0.907	0.365
Baseline Precedent Views (W1)	0.740	0.371	1.992	0.046 *
Political Ideology, liberal	−2.233	2.114	−1.056	0.291
Condition, full	−0.056	0.406	−0.137	0.891
Condition, partial	0.023	0.420	0.054	0.957
Education, some college/associate’s	0.476	0.449	1.060	0.289
Education, four-year degree	−0.824	0.396	−2.081	0.038 *
Education, postgraduate	−0.017	0.338	−0.050	0.961
Gender, woman	−0.236	0.347	−0.678	0.498
Political Knowledge	−1.040	0.828	−1.256	0.209
Court Trust	−0.620	0.260	−2.387	0.017 *
Anti-intellectualism, disagree	−0.762	0.688	−1.107	0.269
Anti-intellectualism, neither agree nor disagree	−0.490	0.591	−0.830	0.407
Anti-intellectualism, agree	−0.923	0.511	−1.805	0.071
Anti-intellectualism, strongly agree	−0.486	0.429	−1.133	0.257
Support federal power, some of the time	0.335	0.792	0.424	0.672
Support federal power, most of the time	0.959	0.593	1.618	0.106
Support federal power, just about always	−0.660	0.361	−1.827	0.068
Readability score	−0.044	0.224	−0.198	0.843
W1 Attitudes: Political Ideology (liberal)	0.495	0.537	0.921	0.357

*Note*. * *p* < 0.05.

**Table 3:** Linear regression model predicting motivated reasoning accuracy scores for *Dobbs*. Scores represent the average of four memory recall items, where higher scores indicate greater recall accuracy and therefore, less evidence of motivated reasoning. Condition, readability and motivated reasoning were measured on Wave 2, while the other controls were measured on Wave 1.

Variable	β	SE	T	*p*
Intercept	1.816	0.560	3.241	0.001 **
Baseline Precedent Views (W1)	0.126	0.112	1.130	0.260
Political Ideology, liberal	0.334	0.618	0.541	0.590
Condition, full	0.196	0.121	1.622	0.106
Condition, partial	0.093	0.131	0.711	0.478
Education, some college/associate’s	−0.033	0.142	−0.233	0.816
Education, four-year degree	−0.164	0.121	−1.355	0.177
Education, postgraduate	−0.049	0.101	−0.487	0.627
Gender, woman	0.181	0.103	1.751	0.082
Political Knowledge	0.415	0.246	1.684	0.094
Court Trust	0.068	0.067	1.026	0.306
Abortion Views, pro-life	0.063	0.141	0.445	0.657
SSI	−0.044	0.075	−0.590	0.556
Readability score	0.029	0.070	0.419	0.676
W1 Attitudes: Political Ideology (liberal)	−0.096	0.155	−0.624	0.533

*Note*. ** *p* < 0.01.

**Table 4:** Linear regression model predicting motivated reasoning accuracy scores for *Loper* scores represent the average of four memory recall items, where higher scores indicate greater recall accuracy and therefore less evidence of motivated reasoning. Condition, readability and motivated reasoning were measured on Wave 2, while the other controls were measured on Wave 1.

Variable	β	SE	z	*p*
Intercept	2.326	0.623	3.733	<0.001 ***
Baseline Precedent Views (W1)	0.064	0.133	0.483	0.630
Political Ideology, liberal	−0.449	0.742	−0.605	0.546
Condition, full	0.004	0.143	0.027	0.978
Condition, partial	−0.088	0.151	−0.582	0.561
Education, some college/associate’s	−0.109	0.171	−0.641	0.522
Education, four-year degree	−0.249	0.145	−1.715	0.088
Education, postgraduate	−0.017	0.122	−0.143	0.886
Gender, woman	−0.095	0.124	−0.767	0.444
Political Knowledge	0.720	0.281	2.557	0.011 *
Court Trust	0.070	0.083	0.835	0.405
Anti-intellectualism, disagree	0.066	0.251	0.262	0.794
Anti-intellectualism, neither agree nor disagree	−0.193	0.215	−0.897	0.371
Anti-intellectualism, agree	0.017	0.180	0.093	0.926
Anti-intellectualism, strongly agree	0.047	0.155	0.301	0.764
Support federal power, some of the time	0.200	0.248	0.805	0.422
Support federal power, most of the time	−0.048	0.180	−0.265	0.791
Support federal power, just about always	0.019	0.119	0.161	0.872
Readability score	−0.075	0.078	−0.964	0.336
W1 Attitudes: Political Ideology (liberal)	0.227	0.187	1.216	0.225

*Note*. * *p* < 0.05; *** *p* < 0.001.

**Table 5:** Linear regression model predicting motivated reasoning evaluation times for *Dobbs*. Time was measured by the number of seconds the participant spent on the task, from their first to last click on the page. Condition, readability and motivated reasoning were measured on Wave 2, while the other controls were measured on Wave 1.

Variable	β	SE	T	*p*
Intercept	36.667	125.431	0.292	0.770
Baseline Precedent Views (W1)	−0.500	25.000	−0.020	0.984
Political Ideology, liberal	99.275	137.419	0.722	0.471
Condition, full	−26.362	26.638	−0.990	0.324
Condition, partial	−57.657	29.024	−1.987	0.048 *
Education, some college/associate’s	−5.703	30.837	−0.185	0.853
Education, four-year degree	3.045	26.677	0.114	0.909
Education, postgraduate	−23.635	22.306	−1.060	0.291
Gender, woman	−16.700	22.899	−0.729	0.467
Political Knowledge	192.086	54.274	3.539	<0.001 ***
Court Trust	23.827	14.770	1.613	0.108
Abortion Views, pro-life	97.993	31.465	3.114	0.002 **
SSI	12.806	16.529	0.775	0.439
Readability score	−18.856	15.386	−1.226	0.222
W1 Attitudes: Political Ideology (liberal)	−12.425	34.442	−0.361	0.719

*Note*. * *p* < 0.05; ** *p* < 0.01; *** *p* < 0.001.

**Table 6:** Linear regression model predicting motivated reasoning evaluation times for *Loper*. Time was measured by the number of seconds the participant spent on the task, from their first to last click on the page. Condition, readability and motivated reasoning were measured on Wave 2, while the other controls were measured on Wave 1.

Variable	β	SE	T	*p*
Intercept	45.417	136.255	0.333	0.739
Baseline Precedent Views (W1)	20.341	29.072	0.700	0.485
Political Ideology, liberal	59.584	162.178	0.367	0.714
Condition, full	66.154	31.291	2.114	0.036 *
Condition, partial	−1.457	32.841	−0.044	0.965
Education, some college/associate’s	14.874	37.919	0.392	0.695
Education, four-year degree	−7.434	32.238	−0.231	0.818
Education, postgraduate	2.426	26.705	0.091	0.928
Gender, woman	−2.309	27.316	−0.085	0.933
Political Knowledge	96.150	61.734	1.557	0.121
Court Trust	28.718	18.215	1.577	0.116
Anti-intellectualism, disagree	−110.960	54.913	−2.021	0.045 *
Anti-intellectualism, neither agree nor disagree	−28.602	47.167	−0.606	0.545
Anti-intellectualism, agree	−16.188	39.376	−0.411	0.681
Anti-intellectualism, strongly agree	−20.328	33.687	−0.603	0.547
Support federal power, some of the time	−2.599	57.197	−0.045	0.964
Support federal power, most of the time	29.932	41.870	0.715	0.476
Support federal power, just about always	−63.775	26.860	−2.374	0.019 *
Readability score	−0.084	17.055	−0.005	0.996
W1 Attitudes: Political Ideology (liberal)	−25.682	40.895	−0.628	0.531 *

*Note*. * *p* < 0.05.

**Table 7:** Contingency table for argument strength for the *Dobbs* case.

	Not at All Strong	Somewhat Strong	Moderately Strong	Very Strong	Extremely Strong	Sum	Percent (Extremely/Very Strong)
**Conservative**	8	14	28	35	30	115	57%
**Liberal**	67	23	19	11	4	124	12%
Sum	75	37	47	46	34	239	
χ^2^ = 82.508, *p* < 0.001							

**Table 8:** Contingency table for argument strength for the *Loper* case.

	Not at All Strong	Somewhat Strong	Moderately Strong	Very Strong	Extremely Strong	Sum	Percent (Extremely/Very Strong)
**Conservative**	4	23	43	31	14	115	39%
**Liberal**	24	30	34	25	7	120	27%
Sum	28	53	77	56	21	235	
χ^2^ = 19.141, *p* < 0.001							

## Data Availability

Data can be accessed through the project’s OSF page: osf.io/2q3mz.

## Supplementary Materials

The following supporting information can be downloaded from https://www.mdpi.com/article/10.3390/bs16071087/s1.

## Abbreviation

The following abbreviation is used in this manuscript:

SSI	Separate Spheres Ideology
