# Supplementary material for: How People Reason About Stare Decisis: The Role of Political Ideology
Source: Behav Sci (Basel). 2026 Jul 1;16(7):1087. doi: 10.3390/bs16071087 (PMC13405648; doi:10.3390/bs16071087)
Supplement: Supplementary file 1 [file behavsci-16-01087-s001.zip › behavsci-4275554-supplementary.pdf]

Supplemental Materials: Saks and Borgida

Table of Contents:

Case summary: Chevron….. p.1
Case summary: Dobbs……..p.2
Precedent definitions……….p.3
Pre-test measures…………. pp.4-6
Wave 1 measures…………..pp.7-11
Wave 2 measures…………..pp.12-15
Pre-test 1 Codebook……….pp.16-19
Attrition analyses…………pp.20-21
VIF tables…………………..pp. 22

Case summary: Chevron

Now, we'd like you to read about a recent U.S. Supreme Court decision and answer a few questions about it.

LOPER BRIGHT ENTERPRISES, ET AL., PETITIONERS V. GINA RAIMONDO, SECRETARY OF COMMERCE, ET AL. (2023)
The Magnuson-Stevens Fishery Conservation and Management Act was signed into law in 1976 and required fisheries to allow federal monitors onboard ships. In 2020, an amendment was submitted and accepted by the agency, the National Marine Fisheries Service (NMFS), which began requiring that fisheries cover the costs of taking federal monitors on fishing trips. Recently, commercial fishing companies sued, arguing that they should not be required to pay those fees, but a federal judge ruled that the NMFS agency could legally impose costs under what is known as the Chevron Doctrine.

The Chevron Doctrine was established in 1984 and says that if Congress is clear when creating a law, courts must adhere to the interpretation of Congress. But if the intent of Congress is unclear, then courts should listen to the agencies' (such as the National Marine Fisheries Service's) interpretations and the experts employed in those agencies.

In 2024, with its decision in Loper Bright, the U.S. Supreme Court struck down the Chevron Doctrine, a decision that breaks with 40 years of precedent. This decision means that the federal courts, instead of agencies, now will need to review the science behind federal policies and regulations more regularly, as companies are expected to challenge the science behind federal regulations more frequently.

Those people who support the decision say it gives more power to federal judges and puts the judicial branch on more equal footing with federal agencies. Others, however, are concerned that protections for consumers, employees, regulation of tech policy and environmental issues may be negatively affected.

Case summary: Dobbs

Now, we'd like you to read about a recent U.S. Supreme Court decision and answer a few questions about it.

DOBBS, STATE HEALTH OFFICER OF THE MISSISSIPPI DEPARTMENT OF HEALTH, ET AL. v. JACKSON WOMEN'S HEALTH ORGANIZATION ET AL. (2021)
Mississippi law states that, "[e]xcept in a medical emergency or in the case of a severe fetal abnormality, a person shall not intentionally or knowingly perform . . . or induce an abortion of an unborn human being if the probable gestational age of the unborn human being has been determined to be greater than fifteen (15) weeks."

Jackson Women's Health Organization challenged this law, arguing that it goes against the precedent that was decided by Roe v. Wade (1973) and supported again in Planned Parenthood of Southeastern Pa. v. Casey (1992), protecting the right to have an abortion. Those who support the precedents of Roe and Casey (those who argued on behalf of Jackson Women's Health Organization) believe that there is a constitutional right to abortion and it should be protected by the federal government. Those with the opposite opinion think the U.S. Constitution does not say anything about abortion and the issue should be left for the state governments to decide.

In 2021, the Court sided against Jackson Women's Health Organization by deciding that the Constitution cannot be interpreted as giving people the right to have abortions and returned abortion policy to the states. This decision overturned the earlier Roe and Casey decisions, overturning a precedent that has stood for nearly five decades.

Since the ruling, at least 16 states have enacted near-total or total abortion bans and over 41 states total have some sort of restriction on abortion. Several states also have introduced legislation for parental consent for abortions and restricting access to abortion via prescribed pharmaceuticals.

Baseline definition:

> "We are interested in learning about your views about the concept of precedent in legal decision making. Please read this definition and answer the following question about your attitudes toward this idea.
>
> Precedent is a principle of the American legal system that says when an existing law is challenged, the Supreme Court should strongly favor honoring past decisions and overrule only if there is an extraordinary reason to do so."

Full **precedent summary:**

"Precedent is a bedrock principle of the American legal system. When an existing law is challenged in the courts, judges should almost always rely on precedent to make their decision."

Partial **precedent summary:**

"Precedent is the starting point and is supposed to be a strong factor in judicial decision making. But there might be times when judges decide to overrule a precedent after considering other factors. The Court can always reverse its decisions. It happens."

Pretest Study Measures:

Attention Check:

- Question: People are very busy these days and many do not have time to follow what goes on in the government. We are testing whether people read questions. To show that you've read this much, answer "extremely interested" and "very interested."
    1. Extremely interested
    2. Very interested
    3. Moderately interested
    4. Slightly interested
    5. Not interested at all

Baseline Attitudes:

- Questions:
    1. In your opinion, how important is it for judges to consider precedent when making decisions? (Response options: Extremely important, very important, moderately important, slightly important, not important at all)
    2. The strength of the law comes from following past precedents (Response options: Strongly disagree, Disagree, Somewhat disagree, Neither agree nor disagree, Somewhat agree, Agree, Strongly agree)
    3. Relying on precedent is crucial to a society like ours that is governed by the rule of law. (Response options: Strongly disagree, Disagree, Somewhat disagree, Neither agree nor disagree, Somewhat agree, Agree, Strongly agree)

Manipulation Check:

1. Question
    - Do you recall reading anything about precedent?
- Answer Choices:
    - (Yes, no, not sure)
2. Question
    - Which of the following <u>best describes</u> your understanding of how the idea of precedent should be used in courts, based on what you just read?
- Answer Choices:
    - (**1**=Precedent is very important and should almost always be upheld unless there is a compelling legal reason not to do so, **2**=Precedent is a starting point, but judges should consider other factors that might have changed since the original law was made, **3**=Precedent doesn't matter much and judges should make decisions based on their own interpretation of the law, **4**=Not sure)

Case Summary:

1. Question
    - How easy or difficult was it for you to read the above paragraph?
- Rating Scale:
    - (**1**=Extremely difficult, **2**=Somewhat difficult, **3**=Neither easy nor difficult, **4**=Somewhat easy, **5**=Extremely easy)
2. Question
    - How legally technical were the above paragraphs?
- Rating Scale:
    - (**1**=Extremely technical, **2**=Somewhat technical, **3**=Moderately technical, **4**=Slightly technical, 5=Not technical at all)
3. Question
    - Would you uphold or overturn precedent in this case?
- Answer Choices:
    - (**1**=Upheld, **2**=Overturned)
4. Question
    - How confident are you about this decision to uphold or overturn precedent?
- Rating Scale:
    - (**1**=Very confident, **2**=Somewhat confident, **3**=Moderately confident, **4**=Slightly confident, 5=Not at all confident)
5. Question
    - Please briefly explain why, and try to be as specific as possible. (open ended)

Political Ideology:

1. Question
    - Participants will be asked a single question asking them to rate how liberal or conservative they are on a seven-point scale.
- Rating Scale:
    - (**1**=Very Liberal, **2**=Liberal, **3**=Slightly Liberal, **4**=Moderate, **5**=Slightly Conservative, **6**=Conservative, **7**=Very Conservative)

Abortion Views:

1. Question
    - With respect to the abortion issue, would you consider yourself pro-choice or pro-life?
- Answer choices:
    - (**1**=Pro-choice, **2**=Pro-life, **3**=Mixed/Neither, **4**=Don't know what terms mean)

Views on federal power:

1. Question
    - How much of the time do you think you can trust the government in Washington to do what is right?
- Rating scale:
    - **(1**=None of the time/Never, **2**=Some of the time, **3**=Most of the time, **4**=Just about always, **5**=Don't know)

Standard Demographics:

1. Gender
2. Education Level
3. Partisanship
    - Rating Scale:
        - (**1**=Strong Democrat, **2**=Weak Democrat, **3**=Independent/lean Democrat, **4**=Independent, **5**=Independent/lean Republican, **6**=Weak Republican, **7**=Strong Republican)
4. Political ideology
    - Rating Scale:
        - (**1**=Very Liberal, **2**=Liberal, **3**=Slightly Liberal, **4**=Moderate, **5**=Slightly Conservative, **6**=Conservative, **7**=Very Conservative)

Final Open-end

1. What do you think is the purpose of this study? Please try to be as specific as possible. (open end)
2. What do you think is the hypothesis being examined in this study? Please try to be as specific as possible.

Wave 1

**case summaries are the same as used in the pre-test study.

Wave 1 Measures:

Attention Check:

- Question: People are very busy these days and many do not have time to follow what goes on in the government. We are testing whether people read questions. To show that you've read this much, answer "extremely interested" and "very interested."
  6. Extremely interested
  7. Very interested
  8. Moderately interested
  9. Slightly interested
  10. Not interested at all

Baseline Attitudes:

- Questions:
  4. In your opinion, how important is it for judges to consider precedent when making decisions? (Response options: Extremely important, very important, moderately important, slightly important, not important at all)
  5. The strength of the law comes from following past precedents (Response options: Strongly disagree, Disagree, Neither agree nor disagree, Agree, Strongly agree)
  6. Relying on precedent is crucial to a society like ours that is governed by the rule of law. (Response options: Strongly disagree, Disagree, Neither agree nor disagree, Agree, Strongly agree)

Manipulation Check:

Question

- Do you recall reading anything about precedent?
- Answer Choices:
  - (Yes, no, not sure)

Question

- Which of the following <u>best describes</u> your understanding of how the idea of precedent should be used in courts, based on what you just read?
- Answer Choices:
  - (**1**=Precedent is very important and should almost always be upheld unless there is a compelling legal reason not to do so, **2**=Precedent is a starting point, but judges should consider other factors that might have changed since the original law was made, **3**=Precedent doesn't matter much and judges should make decisions based on their own interpretation of the law, **4**=Not sure)

Views on federal power

- Question
  - We'd like to ask you a few questions about your beliefs.
  People have different ideas about the government in Washington. These ideas don't refer to Democrats or Republicans in particular, but just to government in general. We want to see how you feel about these ideas.
  How much of the time do you think you can trust the government in Washington to do what is right—just about always, most of the time, only some of the time, or almost never?
- Rating scale
  - **(1**=None of the time/Never, **2**=Some of the time, **3**=Most of the time, **4**=Just about always, **5**=Don't know)

Abortion views

- Question
  - With respect to the abortion issue, would you consider yourself pro-choice or pro-life?
- Answer choices:
  - (**1**=Pro-choice, **2**=Pro-life, **3**=Mixed/Neither, **4**=Don't know what terms mean)

Anti-intellectualism

- I'd rather put my trust in the wisdom of ordinary people than the opinions of experts and intellectuals.
  - Answer choices:
    - Strongly disagree, disagree, neither agree nor disagree, agree, strongly agree
- From what you have heard or read, do you believe increases in the Earth's temperature over the last century are due more to the effects of pollution from human activities or natural changes in the environment that are not due to human activities. Or, you believe that there has been no change in the climate?
  - Answer choices:
    - Human activities, natural changes, there has been no change in the climate
- How much confidence do you have in the scientific community overall?
  - Answer choices:
    - A great deal, only some, hardly any

Separate Spheres Ideology (SSI) Scale
We'd now like to get your opinion on gender issues.

- Women can learn technical skills, but it doesn't come as naturally as it does for most men.
- If one person in a heterosexual marriage needs to quite working, it usually makes more sense for the husband to keep his job
- When it comes to voting for a president, I'm more comfortable trusting a man to make tough political decisions than a woman
- Children with single parents can be just as well off as children with both a mom and a dad
- When a married couple divorces, judges shouldn't assume that the mother is a more "natural" parent.
- Most men naturally enjoy a tough and competitive career more than women do.
- Women can learn how to be good leaders in the workplace, but it doesn't come as naturally as it does for most men.
- It's natural for a woman to be fulfilled by taking care of her children, but most men feel better when they have a good career, too.
- There are certain caregiving jobs, like nursing that just naturally fit with women's skills better than men's skills.
- It's just as important to most women as it is to men to have a successful career.
- When it comes to making tough business decisions, men tend to have special abilities that most women don't have.
- Answer choices:
    - Strongly disagree, disagree, neither agree nor disagree, agree, strongly agree

Trust in the court measures- Malhotra and Jessee (2014)

- Question
    1. The Supreme Court can usually be trusted to make decisions that are right for the country as a whole.
- Rating scale:
    - (**1**=Strongly disagree, **2**=Disagree, **3**=Neither agree nor disagree, **4**=Agree, **5**= Strongly agree)
- Question
    2. The U.S. Supreme Court should have the right to say what the Constitution means, even when the majority of the people disagree with the Court's decision
- Rating Scale:
    - (**1**=Strongly disagree, **2**=Disagree, **3**=Neither agree nor disagree, **4**=Agree, **5**= Strongly agree)
- Question
    3. How much do you approve of the performance of the Supreme Court?

- Rating Scale:
    - (**1**=Strongly disapprove, **2**=Somewhat disapprove, **3**=Somewhat approve, **4**=Strongly approve)
    -

Law Background

-Have you, or any member of your immediate family, ever worked in the legal profession, either as a lawyer, paralegal or in a law office, including a prosecutor's or public defender's office?

-Answer choices: yes, no

-Have you, or any member of your immediate family, had any legal training including law courses, paralegal programs, or on-the-job training?

-Answer choices: yes, no

Political Knowledge

Now, we would like to ask you a few questions about public figures and the political system. Please answer each of the following questions and do not search for answers on the internet.

- What job or political office does JD Vance currently hold?
    - Answer choices: Attorney General, Vice President, Secretary of State, Governor of New Hampshire
- What job or political office does John Roberts currently hold?
    - Answer choices: Secretary of Defense, Attorney General, Senate Majority Leader, Secretary of the Interior, Chief Justice of the Supreme Court
- How long is the term of office for a U.S. senator?
    - Answer choices: 2 years, 4 years, 5 years, 6 years, 8 years
- Whose responsibility is it to nominate judges to the Federal Courts —the President, the Congress or the Supreme Court?
    - Answer choices: The President, Congress, The Supreme Court

Demographics

Political ideology

- Question
    - Participants will be asked a single question asking them to rate how liberal or conservative they are on a seven point scale.
- Rating Scale:
    - (**1**=Very liberal, **2**=Liberal, **3**=Slightly liberal, **4**=Moderate, **5**=Slightly conservative, **6**=Conservative, **7**=Very conservative)

Partisan identity

- Question
    - Participants will be asked a single question asking them to rate how Democratic or Republican they are on a seven point scale.
        - Rating Scale:
            - (**1**=Strong Democrat, **2**=Weak Democrat, **3**=Independent/lean Democrat, **4**=Independent, **5**=Independent/lean Republican, **6**=Weak Republican, **7**=Strong Republican)

Age

- What is your age?
    - Answer choices: 18-29, 30-49, 50-64, 65+

Education

- Which of the following best describes the highest level of education you have achieved?
    - Answer choices: High school or less, Some college/associate's degree, Four-year college degree, Postgraduate degree (Master's, PhD)

Race

- What is your race or ethnicity?
    - Answer choices: White/European descent, Black/African American, Hispanic, Asian/Asian-American, Native American/Indigenous, Mixed-race/Multiracial

Gender

- What is your gender
    - Answer choices: Man, Woman, Non-binary/genderqueer, Another gender not listed, Prefer not to say

Religion

- What is your present religion, if any?
    - Answer choices: Protestant, Roman Catholic, Orthodox Christian, Latter-Day Saints, Jewish, Muslim, Buddhist, Hindu, Atheist, Agnostic, Something else, Nothing in particular

Religiosity

- How important is religion in your life?
    - Answer choices: Not important at all, a little important, moderately important, very important, extremely important

Mother's Education

- What is the highest level of education your mother has completed?
    - Answer choices: Less than high school, high school diploma or equivalent, some college no degree, associate's degree, bachelor's degree, master's degree, professional degree, doctoral degree

Wave 2 Measures:

Full precedent summary, partial precedent summary or no summary

Attention Check:

- Question: People are very busy these days and many do not have time to follow what goes on in the government. We are testing whether people read questions. To show that you've read this much, answer "extremely interested" and "very interested."
    11. Extremely interested
    12. Very interested
    13. Moderately interested
    14. Slightly interested
    15. Not interested at all

Manipulation Check1

Now, we just want to check whether you read and understand what you just read, regardless of your own personal views. If you did not read anything about precedent, just indicate that in your responses.

-Do you recall reading anything about precedent?

-Answer choices: Yes, no, not sure

-Regardless of your own personal views, which of the following best describes your understanding of how the idea of precedent should be used in courts, based on what you just read?

-Answer choices: Precedent tis very important and should almost always be upheld unless there is a compelling legal reason not to do so, precedent is a starting point but judges should consider other factors that might have changed since the original law was made, precedent doesn't matter much and judges should make decisions based on their own interpretation of the law, I did not read anything about precedent

Manipulation Check2

1. In your opinion, how important is it for judges to consider precedent when making decisions? (Response options: Extremely important, very important, moderately important, slightly important, not important at all)
2. The strength of the law comes from following past precedents (Response options: Strongly disagree, Disagree, Somewhat disagree, Neither agree nor disagree, Somewhat agree, Agree, Strongly agree)
3. Relying on precedent is crucial to a society like ours that is governed by the rule of law. (Response options: Strongly disagree, Disagree, Somewhat disagree, Neither agree nor disagree, Somewhat agree, Agree, Strongly agree)

Participant assessment of cases

- How easy or difficult was it for you to read the above paragraph?
  - Answer choices: Extremely difficult, somewhat difficult, neither easy nor difficult, somewhat easy, extremely easy
- How legally technical for you were the above paragraphs?
  - Answer choices: Extremely technical, somewhat technical, moderately technical slightly technical, not technical at all
- How strong do you think the winning argument in this case was?
  - A note of clarification will be added to the question stem for each case.
  - For Dobbs: Note: The winning argument was that there is no constitutional right to abortion.
  - For Loper Bright: Note: The winning argument was that courts should have more power to interpret laws compared to federal agencies.
  - Answer choices: Not at all strong, somewhat strong, moderately strong, very strong, extremely strong, not sure
- Had it been up to you, would you have upheld or overturned precedent in this case?
  - A note of clarification will be added to the question stem for each case.
  - For Dobbs: Note: Upholding precedent means upholding the precedent from Roe v. Wade and keeping a constitutional right to abortion.
  - For Loper Bright: Note: Upholding precedent means upholding the Chevron doctrine and allowing federal agencies more power to interpret laws.
  - Answer choices: I would have upheld precedent in this case, I would have overturned precedent in this case
- Which of the following is the main reason for your decision?
  - Answer choices (Dobbs): I believe it should be up to the states to decide rather than the federal government, I generally favor upholding precedent, I believe the choice about whether to have an abortion should be decided by the person seeking an abortion, Other reason
  - Answer choices (Chevron): I don’t believe the government should require the fishing companies to pay for the monitors, I generally favor upholding precedent, I am concerned about the effect this will have on the balance of power in the government, I support having more regulations about the environment and other issues, I could not understand the passage very well, Other reason
- Please elaborate on your reasoning, and try to be as specific as possible. [open end]
- How confident are you about your decision to uphold or overturn precedent?
- Answer choices: Not at all confident, Slightly confident, Moderately confident, Very confident, Extremely confident
- Timer (records how long participant spends on the page)

Case Familiarity

- How familiar were you with the Dobbs v. Jackson Women's Health Organization case, before you read about it just now?

-Answer choices: Not familiar at all, slightly familiar, moderately familiar, very familiar, extremely familiar

- How familiar were you with the Loper Bright Enterprises v. Raimondo case, before you read about it just now?

-Answer choices: Not familiar at all, slightly familiar, moderately familiar, very familiar, extremely familiar

Evidence of Motivated Reasoning

- Question
    - Instructions: Please read the following statements and indicate whether you think they are TRUE or FALSE based on what you read earlier.
    - The Dobbs v. Jackson Women's Health Organization case overturned a precedent. (TRUE)
    - The Dobbs case was related to a precedent that has existed for three decades. (FALSE, the vignette said it stood for 50 years)
    - There was no existing precedent that judges used to decide Dobbs. (FALSE, there was precedent from Roe and Casey)
    - The Dobbs decision decided that the Constitution can NOT be interpreted as giving people the right to have abortions. (TRUE)
    - The Court decided to overturn the Chevron doctrine. (TRUE)
    - The Chevron doctrine was established in the last 10 years. (FALSE).
    - The *Loper Bright Enterprises v. Raimondo* and *Relentless, Inc. v. Department of Commerce* cases have to do with an existing precedent (the Chevron doctrine) (TRUE).
    - The decisions in the cases *Loper Bright Enterprises v. Raimondo* and *Relentless, Inc. v. Department of Commerce* are expected to give more power to federal agencies. (FALSE)
- Rating Scale:
    - (**1**=TRUE, **2**=FALSE)

Feedback
Finally, we'd like to get your feedback on this survey.

- What do you think is the purpose of this study? Please try to be as specific as possible. [open end]

- What do you think is the hypothesis being examined in this study? Please try to be as specific as possible. [open end]

Pretest 1 Codebook

Up to three codes could be assigned to an open-ended response, in which case they would be listed in the order they appear. 99 was only coded if no other codes fit.

Code	Values	Description	Examples
Consistency	1= Consistent<br>2= Not consistent<br>99= Unable to code	Whether the response is consistent with the reported decision to uphold or overturn in the earlier question	
Specificity	1=Specific<br>2=Generic/global attitude<br>99=Unable to code	Whether the response given is a specific reason(s) or more of a general/broad attitude	
Thematic codes (Dobbs)	1= reference to abortion identity<br>~~2=general views about abortion~~<br>3=discussion of precedent, positive<br>4=discussion of precedent, negative<br>5=reference majority opinion<br>6=states' rights (positive or negative)<br>7=personal choice, general abortion views, government and other entities have no right to tell people what to do<br>8=references to constitution<br>9=concern for children's welfare, either pro-choice or pro-life<br>10=moral beliefs<br>11=Supreme Court bias<br>12=Concern for Mother's health or welfare, whether pro-choice or pro-life<br>98=other reason given	Which of the themes listed best describes the response	1 "I am pro choice so I would want to uphold the Roe V Wade precedent."<br>2 "I strongly feel it is the right of a woman to decide what to do with her body."<br>3 "In this case, no new information, technology, or subject matter has been introduced to the earlier precedent so therefore it should be upheld as it has been in the past"<br>"Legal decisions should always consider precedence"<br>4 "I feel that Jackson Women's Health organization should have brought more reasons with them. The fact that they only provided prior decisions and a statement that it is unconstitutional does not sway me."<br>5 "The idea of precedent is that you continue with the past rulings and stay consistent to past actions of that law. Laws have to much up for interpretation and precedent is what allows them to not be abused. Also with so much of public opinion supporting to

	99=Don't know/nothing more to add/no response/no theme		uphold, I would respect majority opinion too."<br>6 "I think it should be left up to the states decide because it isn't in the constitution"<br>7 "I agree that abortion decisions should not be left up to a government or anyone besides oneself"<br>"If someone wants to have an abortion, I don't see why anyone else cares."<br>8 "I agree that the Constitution cannot be interpreted as giving people the right to have abortions (I would need to see the exact section they're referencing) and therefore don't think the concept of precedence necessarily applies here."<br>9 "I believe babies that have a heartbeat deserve their life."<br>"I am very much so PRO Life. I believe that abortion is murder,and if the women that are pregnant don't want there child then there are so many couples that can't have children that would love to adopt from a unwanting mother."<br>10 "I would choose to overturn based on scientific evidence of infant vitality, ability to feel pain, and morality behind abortions."<br>"There was a reason for this precedent, and to overturn it is not only morally wrong, but also disrespectful."<br>"Because it is important that young girls should have a decision on keeping a baby or not."<br>11 "I feel the Supreme Court in this instance was not taking past precedent as defined earlier in this study into account. That they were acting on their personal beliefs on how they wanted the law to be and working their way backwards from there."

			12 "I believe that if there is some difficulty with the pregnancy such as an anomaly or if the pregnancy has been deemed not viable, the decision to abort should be made by the woman and her doctor."
Thematic codes (Chevron)	1=too difficult to read/didn't understand the passage because it got cut off, so can't say<br>2=overturn because the government should not require monitors and require fishers to pay for them<br>3=discussion of precedent, positive<br>4=discussion of precedent, negative<br>5=Federal government/Congress should stay out of this issue entirely<br>6=references the environment<br>7=views on governmental structure<br>8=power of agencies (positive or negative)<br>9=references science<br>98=other reason given<br>99=Don't know/nothing more to add/no response/no theme		1 "I honestly have no idea what I read about"<br>2 "With that said, I don't believe these companies should have to front the cost of this."<br>"The monitors should be provided"<br>3 "The courts previously believed that it was okay to let agencies interpret the laws, so I would refer back to that precedent."<br>4 "My personal opinion is laws in general should be reviewed every 20 years "<br>5 "I do not think that the federal government needs to be in charge of everything."<br>6 "I believe that we should be protecting and putting regulations forward to protect the environment"<br>7 "Overturning Chevron appears to be a deliberate attempt to upset the balances of power that are supposed to exist in our government."<br>8 "I believe that agencies have too much unchecked power"<br>9 "I feel the courts will be better able to examine the science and reasoning behind agency regulations, making policies more transparent and accountable."
Suspected Chat GPT/AI	1=not AI<br>80=AI	Whether coder suspects response comes from Chat GPT/AI	

OPEN END 3: Participants were asked, at the end of the survey, to guess what the <u>purpose</u> of the study was

Code	Values	Description	Examples
Identification of study purpose	1=unable to identify purpose<br>2=able to partly or mostly identify study purpose<br>3=completely identified study purpose<br>99=unable to code	Whether participants correctly identified the purpose of our study	

OPEN END 4: Participants were asked, at the end of the survey, to guess what the hypotheses of the study were

Code	Values	Description	Examples
Identification of study hypotheses	1=unable to identify hypothesis<br>2=able to partly or mostly identify study hypothesis<br>3=completely identified study hypothesis<br>99=unable to code	Whether participants correctly identified the hypotheses	

Attrition comparisons

Baseline views toward precedent by whether or not participants dropped out after Wave 1

```
Wilcoxon rank sum test with continuity correction

data:  baseline_w1 by dropout
W = 1323, p-value = 0.8901
alternative hypothesis: true location shift is not equal to 0
```

Political knowledge average by whether or not participants dropped out after Wave 1

```
Wilcoxon rank sum test with continuity correction

data:  polknow_average by dropout
W = 1939, p-value = 0.07993
alternative hypothesis: true location shift is not equal to 0
```

SSI average by whether or not participants dropped out after Wave 1

```
        Wilcoxon rank sum test with continuity correction

data:  SSI_average by dropout
W = 1713.5, p-value = 0.5324
alternative hypothesis: true location shift is not equal to 0
```

Ideology by attrition group

	**No**	**Yes**
Very liberal	42	2
Liberal	62	4
Slightly liberal	22	0
Moderate	16	1
Slightly conservative	25	2
Conservative	58	2
Very conservative	33	1

Age by attrition group

	**No**	**Yes**
18-29	62	1
30-49	125	9
50-64	48	2
65+	23	0

Education by attrition group

	**No**	**Yes**
High school or less	20	2
Some college/associate’s degree	57	4
Four-year college degree	117	4
Postgraduate degree (Master’s, PhD)	64	2

VIF Tables

Dobbs

	GVIF	Df	GVIF^(1/(2*Df))
baseline_w1	1.403973	1	1.184894
ideo2	52.658246	1	7.256600
condition	1.179496	2	1.042135
Education	1.467120	3	1.065968
binarygender	1.146151	1	1.070584
polknow_average	1.312739	1	1.145748
courttrust_average	1.330564	1	1.153501
abortionID	1.165544	1	1.079604
SSI_average	1.212809	1	1.101276
dobbs_readable	1.148888	1	1.071862
baseline_w1:ideo2	54.087242	1	7.354403

Loper

	GVIF	Df	GVIF^(1/(2*Df))
baseline_w1	1.403973	1	1.184894
ideo2	52.658246	1	7.256600
condition	1.179496	2	1.042135
Education	1.467120	3	1.065968
binarygender	1.146151	1	1.070584
polknow_average	1.312739	1	1.145748
courttrust_average	1.330564	1	1.153501
abortionID	1.165544	1	1.079604
SSI_average	1.212809	1	1.101276
dobbs_readable	1.148888	1	1.071862
baseline_w1:ideo2	54.087242	1	7.354403
